# How well are Swiss French physicians prepared for future practice in primary care?

**DOI:** 10.1186/s12909-018-1168-4

**Published:** 2018-04-04

**Authors:** N. Junod Perron, M. C. Audetat, S. Mazouri, M. Schindler, D. M. Haller, J. Sommer

**Affiliations:** 1 Institute of Primary Care, Geneva University Hospitals, 22 av Beau-Séjour, 1211 Genève 4, Switzerland; 2 Unit of Development and Research in Medical Education, Geneva Faculty of Medicine, Geneva, Switzerland; 3 Unit of Primary Care, Geneva Faculty of Medicine, Geneva, Switzerland; 4 Division of Primary Care, Geneva University Hospitals, Geneva University Hospitals, Geneva, Switzerland

**Keywords:** Primary care, Preparedness, Transition, Graduate training, Independent practice

## Abstract

**Background:**

Moving from postgraduate training into independent practice represents a major transition in physicians’ professional life. Little is known about how Swiss primary care graduates experience such a transition. The aim of this study was to explore the extent to which primary care physicians who recently set up private practice felt prepared to work as independent practitioners.

**Methods:**

We conducted 7 focus groups among recently established (≤ 5 years) primary care physicians in Switzerland. Questions focused on positive and negative aspects of setting up a practice, and degree of preparedness. Transcripts were analysed according to organisational socialisation and work role transition frameworks.

**Results:**

Participants felt relatively well prepared for most medical tasks except for some rheumatologic, minor traumatology, ENR, skin and psychiatric aspects. They felt unprepared for non clinical tasks such as office, insurance and medico-legal management issues and did not anticipate that the professional networking outside the hospital would be so important to their daily work. They faced dilemmas opposing professional values to the reality of practice which forced them to clarify their professional roles and expectations. Adjustment strategies were mainly informal.

**Conclusion:**

Although the postgraduate primary care curriculum is longer in Switzerland than in most European countries, it remains insufficiently connected with the reality of transitioning into independent practice, especially regarding role development and management tasks. A greater proportion of postgraduate training, with special emphasis on these issues, should take place directly in primary care.

## Background

Primary care is the provision of integrated, accessible health care services by clinicians who are accountable for addressing a large majority of personal health care needs, developing a sustained partnership with patients and practicing in the context of family and community [1, 2].

Postgraduate training in primary care varies according to countries and health systems. Vocational training in general practice training is required in the UK since 1976 [3]. Specific training in general practice in all other EU countries has only been required since 1995 and should include at least 50% vocational training [4]. In 2004, a majority of EU countries offered at least one year of training in general practice with 1 to 3 years of hospital training [5]. In the United-States, training in primary care mainly takes place in hospitals with continuity clinics’ half day activities during the three years [6]. In Switzerland, postgraduate trainees in primary care are required to train for a minimum of 6 months in ambulatory medicine and two years in hospital medicine as part of a five-year postgraduate curriculum in general internal medicine [7]. The 6 months training in ambulatory medicine can take place either in academic primary care clinics or private practices.

Doctors experience many transitions through their professional life for which they often feel under-prepared [8–11]. They frequently develop high stress and negative emotions during these transitions that can impact on both doctors’ performance and patient safety [12, 13]. Transition is defined as a process of change in which “individuals experience a personal awareness of discontinuity in their life space, forcing them to learn and adapt to new tasks and roles in the new situation” [14, 15]. Moving from training to independent practice represents a transition for which physicians may feel more or less prepared. Preparedness refers to “a feeling of confidence or self-belief, in carrying out procedural or communication tasks, dealing with particular clinical situations or making clinical judgements” [16, 17]. It requires both confidence and ability to adapt to future work [17]. Theoretical frameworks describing work-role transitions and organizational socialization state that people face new affective states and identity changes [14, 18–20]. They have to develop a sense of competence, they need to clarify their role and form new interpersonal relationships to adapt to the new setting [18, 20]. The degree of stress experienced during work transitions seems higher if there is a mismatch between pre-expectations and the actual professional conditions. Prior work experience in a similar setting tends to facilitate the development of realistic expectations regarding a job [18]. “On the job” social support from senior peers reduces such stress and leads to positive adjustment outcomes [19]. It suggests that both anticipation of roles and tasks of the new job and appropriate social support during the transition facilitate skill transfer and autonomy.

This transition period at the end of postgraduate training has been studied in various ways. Some surveyed physicians from different specialities on the mastery of different medical and/or non medical tasks as they switched from a registrar to a consultant position [21, 22], acquired a title of specialist [23] or moved from a status of primary care trainee to that of primary care practitioner [24, 25]. Others, mainly UK studies, explored physicians’ perceptions on such transition through semi-structured interviews or focus groups [17, 26, 27]. In the field of primary care, most surveyed or interviewed physicians felt insufficiently trained for some medical tasks, poorly equipped for self-employment issues such as taxation, pensions, employment law as well as for practice management and leadership skills [25, 28]. They also reported a sense of loneliness, unpreparedness for self-care and for the emotions related to this transition period, and difficulties finding a balance between life and work [27, 28].

Little is known about how well doctors having a mainly hospital-based training are prepared to work as primary care physicians. In Switzerland, the training is longer, less practice oriented, flexible and not under the responsibility of medical schools. The fact that physicians in training are free to choose their training places as long as they fulfill the criteria of the Swiss federation of physicians (FMH) might make the transition to private practice even more difficult by lack of anticipation and planning.

The aim of this study was to explore the extent to which primary care graduates transitioning into primary care practice felt prepared to perform as independent doctors in Switzerland. In order to improve the current curriculum and fill the gap between postgraduate training and professional life, we were especially interested in exploring the range of tasks/dimensions for which they felt prepared or not, the emotional stress and tensions they experienced, and the processes used or needed to prepare and adjust to their new role as independent practitioners.

## Methods

### Setting and participants

The study was carried out among primary care physicians working in the French-speaking part of Switzerland (which represents a fourth of the Swiss population). Inclusion criteria were: certified training in general internal medicine (FMH) and newly established in a primary care practice in the past five years. The list of all potential participants was obtained through the medical societies and public health services of the cantons of Geneva, Vaud, Neuchâtel and Fribourg and through the head of primary care physicians’ association for Valais. For two cantons with a lower density of physicians (< 210/100000 inhabitants: Neuchâtel, Fribourg), all physicians were contacted. For cantons with a higher density of physicians (> 210/100000 inhabitants: Geneva, Vaud), the sample of physicians was selected in order to include a representative variety of practice settings (size and location of the practice). In Valais, only a selected list of physicians could be contacted. A research assistant made contact with the primary care physicians (by email and/or by phone) and invited them to participate.

### Data collection

A total of seven focus groups were conducted: three in Geneva, one in Vaud, one in Neuchâtel, one in Fribourg and one in Valais. 35/118 contacted physicians agreed to participate (Table 1).

**Table 1:** Number of physicians who participated out of the number meeting the selection criteria

	Number of physicians meeting selection criteria	Number of physicians who did not answer	Number of physicians who declined^a^	Number of physicians who agreed to participate	Number of physicians who finally participated
Geneva	80 (66 contacted)	38	10	18	12
Vaud	43 (31 contacted)	17	10	4	4
Neuchâtel	27 (all were contacted)	11	12	4	4
Fribourg	9 (all were contacted)	3	2	4	4
Valais	No data	No data	No data	5	5

^a^Reasons for declining: lack of availability, lack of interest, maternity leave

### Procedure

The focus group guide was pre-tested in an initial focus group discussion with physicians working in the Primary Care Unit at the University of Geneva (Table 2). Questions focused on participant’s perceptions regarding their preparedness in carrying out the tasks required for independent primary care practice.

**Table 2:** Interview guide for the focus groups

1. If you think back at setting up your practice, what were the
a. Positive aspects?
b. Negative aspects?
2. For which aspects did you feel well prepared?
3. For which aspects did you feel poorly prepared?
If you look back on your postgraduate training:
4. What was useful to perform well in practice?
a. Where did you learn it?
5. What did you miss to perform well in practice?
a. How did you remediate this?
6. If something had to be changed in the postgraduate training in order to better prepare you for the practice, what would be your wishes?

Each focus group lasted 90–120 min and took place in one of the participants’ office in each canton. They were conducted by two facilitators who had no past or current hierarchy link with the participants in order to ensure that participants felt free to express their views on how well their postgraduate training prepared them for the practice. Discussions were audiotaped and field notes were written immediately following each session. Audiotapes were transcribed ad verbatim.

The study was granted a waiver from approval by the Ethical Committee of the canton of Geneva. Approval by the Ethical Committee is not necessary under Swiss law on research for studies in which non personal health-related data are collected [29]. Participants signed a consent form and were informed that the data would be analysed anonymously.

### Analysis

Analysis of transcripts followed the “editing style” approach described by Miller and Crabtree [30]. Investigators included two experienced clinical teachers working in an academic primary care division (NJP and DMH), a professor of primary care medicine and experienced primary care practitioner (JS), a resident having trained and worked in a private practice and an academic primary care division (SM), a specialist in medical education (MCA) and a sociologist (MS). All of them read 3 transcripts from different geographical areas and independently identified key themes and passages. After discussion, an initial list of codes was developed, including themes such as type of training, working context, acquired and missing competencies, values/goals, role, professional relationships, physician-patient relationships, coping strategies and emotions. Perceptions regarding preparedness were then reorganized according to the main dimensions of theoretical frameworks about organizational socialization and work role transitions [14, 18–20]. The following categories were chosen: - performance (including tasks/competencies); − interpersonal relationships including physician-patient and inter-professional relationships; − goals and values; −roles; and finally -adjustment strategies in relation to all these professional dimensions. NJP’s coding of four focus groups were cross-checked by 3 co-investigators (SM, MS, MCA) and required only minor changes. Therefore, coding consensus was considered to be strong and NJP coded the remaining focus groups. Point of saturation was reached after five focus groups but seven focus groups were conducted in order to include participants from all the different cantons. Transcripts were coded using Maxqda software for qualitative data analysis [31]. All translations of participants’ utterances are the authors’.

## Results

Out of the 35 physicians who agreed to participate, 29 physicians finally took part in the study. Three focus groups took place in Geneva (*n* = 12; mean age 40.50 (SD 4.52), 66.% women), 1 in Vaud (*n* = 4; mean age 44.25 (SD 4.52), 100% women), 1 in Neuchatel (n = 4; mean age 42.5 (SD 6.85), 25% women), 1 in Fribourg (n = 4; mean age 38.5 (SD 3.87), 25% women), and 1 in Valais (*N* = 5; mean age 41.20 (SD 2.56); 60% women).

Participants had completed most of their training in Switzerland and spent almost 10 years in training (more than half in hospital training) before entering in private practice (Table 3). With regards to ambulatory training, a third had trained exclusively in an academic setting, 27% combined academic and private practice while 20% combined academic and medico-surgical training. They currently worked mostly in an urban or suburban area and predominantly in small size group practices.

**Table 3:** Participating physicians’ socio-demographic characteristics

Sociodemographic and clinical data	*N* = 29
Women n(%)	17 (58.6)
Mean age (SD)	41.1 (4.39)
Pre-graduate training in Switzerland n(%)	25 (86.2)
Graduate training in Switzerland n(%)	27 (93.1)
Years of post-graduate training mean (SD)	9.34 (6.79)
Years of post-graduate training in hospital settings mean (SD)	6.79 (2.94)
Years of post-graduate training in ambulatory settings mean (SD)	3.80 (3.16)
Type of training in ambulatory setting n(%)	
- Academic outpatient clinic in general internal medicine	10 (34.5)
- Private practice	2 (6.9)
- Medico-surgical center	1 (3.4)
- Academic clinic and private practice	8 (27.6)
- Academic clinic and medico-surgical center	6 (20.7)
- Academic clinic, private practice and medico-surgical center	1 (3.4)
- other	1 (3.4)
Years in private practice mean (SD)	3.03 (1.42)
Geographical location n(%)	
- urban	14 (48.3)
- suburban	12 (41.4)
- rural	3 (10.3)
Type of office n(%)	
- solo	2 (6.9)
- -2-4 colleagues	18 (62.1)
- > 4 colleagues	9 (31.0)

**Fig. 1:**
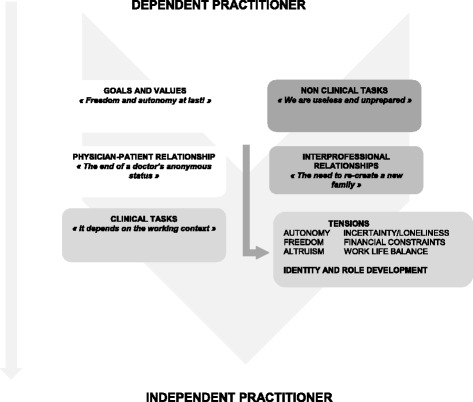
Work dimensions affected by the transition from dependent to independent primary care practice

Figure 1 summarizes the main work dimensions reported by participants (Fig. 1).

Shifting from dependent to independent practice was experienced as a major transition for which participants reported contrasting perceptions. On one hand, the transition was described as a major source of stress and anxiety, «*the unknown and a cosmic vacuum*».

On the other hand, it was perceived as an accomplishment and the end of a long journey allowing them to be themselves, «*the icing on the cake».*

Perceptions varied according to the type of work dimensions they discovered and the type of training and working context they experienced.

### Goals and values: Freedom and autonomy at last!

The most positive elements referred to the values and goals participants associated with their status of independent practitioner: feelings of freedom, autonomy and mastery over time, schedules, space and actions which contrasted positively with institutional work where participants felt they consistently had to fit into the mould. Such changes favourably influenced the work/life balance of both male and female participants.

*«I would say, among the positive things when setting up a private practice, it is clearly the freedom of practice, the ability to master what I want to do, to do something when I want» Focus group (FG)1 line 138*

*«To be on a sailing boat, at the speed we want» FG5 line 546*

However, this was especially the case for female doctors who struggled to combine training and family life in environments where full time jobs were imposed.

*« I am glad I left the hospital and went into private practice, it is clear that I have much more time for myself, my family. It is easier to manage this balance between professional and family life.» FG1, line 381*

### Patients: The end of doctors’ anonymous status

Participants reported having discovered many positive elements related to patients. First, patients attending private practices were considered to be “easier” and more diverse than those encountered in academic outpatient training clinics which often provide care to vulnerable and precarious patients.

*«It is true that at the XXX (academic outpatient clinic), when I trained, it was rather complicated because there were undocumented immigrants, asylum seekers, patients who did not speak French, clinical encounters were rather difficult. It is true that once we go through this experience, everything seems easier elsewhere» FG2 line 752*

Feeling that patients had chosen them as “their doctor“and that they could also choose their patients was a very positive experience. The emotional bond they felt developing on both sides was very rewarding but was often linked with a sense of responsibility and a risk of emotional overload.

*«It is the end of the anonymous status of the hospital doctor, we are recognized as Dr. X… and people come to see Dr X because they were told to do so…» FG4 line 22*

### Medical tasks: It depends on professional context

Participants generally felt adequately prepared to perform most medical tasks in the field of internal medicine. They enjoyed the diversity of the medical problems they faced.

*«What is nice in practice is to have (someone with) a small urinary tract infection, followed by a difficult case, then an alcohol dependent patient. All this mixture» FG4 line 294*

They felt uncomfortable, however, dealing with common problems in rheumatology/sports medicine, minor traumatology, Ear-Nose-Throat (ENT), dermatology and psychiatry, whatever their professional context.

*«Rheumatology, skin disease, paediatrics, these are the three rotations I should have done… these are things we see all the time… I do not feel comfortable to constantly refer them or to say to the patient … I don’t know, I don’t’ know, I don’t know» FG2 line 620*

They considered that paediatrics, gynaecology, as well as surgery skills were crucial for those working in rural areas. It was especially the case for participants who had not planned their training according to their current rural practice context.

Lacking knowledge or having to make decisions to refer, treat or wait in the absence of supervisors was experienced by some physicians as uncomfortable, especially for those who had no experience of outpatient care outside an emergency department or were currently working in solo practices.

Most participants favored a combination of both private practice and academic primary care training but there was no consensus on the timing of training beside the need to train first within a hospital. Training in an academic primary care clinic was considered to favor knowledge in Evidence Based Medicine, psychosocial complexity and chronic diseases. Training in private practice allowed physicians to be exposed to a larger case mix of patients and clinical problems, develop more autonomy and become more familiar with office management.

### Inter-professional relationships: The need to create a new “family”

Entering a large group structure was perceived by some participants as being very comfortable, because it replicated a mini hospital team with facilitated access to specialists. Several participants reported not having anticipated the importance of having a network of specialists once in independent practice. This was especially challenging when physicians opened a practice in an area where they did not train and missed the informal network of colleagues they had encountered in training institutions.

*«To find yourself alone in your office…. It was very very difficult. Suddenly, we no longer belong to this large family, and we have to create our “own new family”, let’s say » FG1 line 588*

However, some reported a positive change in power distribution/relationship when interacting with specialists in independent practice: they perceived specialists to be much more friendly and collegial than in institutional settings, feeling that independent specialists relied on them to develop their own patient population.

*«There is this harshness of the hospital environment that does not exist outside… outside hospital, specialists depend on us. It is fascinating to see that specialists who are a little straightforward at the beginning, they step back because they find themselves without patients anymore» FG5 line 331*

### Non clinical tasks: We are useless and unprepared

The most negative perceptions related to non-clinical tasks for which all participants felt both incompetent and unprepared by lack of training or absence of concrete guidelines. They were two generic tasks they did not anticipate as they started their practice: “*pre-installation*” itself such as taking out personal insurances, organising a pension plan, preparing a business plan, choosing the right electronic health record or billing software and - “*practice*” management such as administrative, financial and human resources management tasks.

*«When we have to manage staff, we are useless, objectively useless! » FG3 line 243*

*« I find myself catapulted as a manager, without having ever studied anything related to the management of a small company… personally, I have no interest in it but the problem is that we have to do it» FG7 line 103*

Schedule and patient flow management was an additional unexpected source of stress for daily work. Finally, dealing with medico-legal or medico-social issues such as completing reports for health insurances or legal institutions was also described as a rather new and challenging task.

*«It is sometimes extremely difficult to manage work capacity, sickness-leave certificates, their duration,… we find ourselves experts for patients that we do not know well, we have not been well trained to do it… we “guesstimate” » FG4 line 382*

### Role and identity development

The reality of daily practice obliged them to reconsider or clarify their roles as independent practitioners. There was a tension between personal values such as autonomy and feelings of uncertainty and loneliness regarding medical decisions.

*“We know nothing about almost everything. We will never know how to care for everything” FG7 line 712*

Sense of freedom, community engagement and altruism were limited by the need to earn a living and cope with financial and administrative pressures and constraints.

*« I decided to study medicine in order to help others, I never raised the issue of money, it is something that I basically denigrate…I have no interest in the business volume and profitability, we are forced to get interested in something for which we have no interest » FG5 line 175*

Finally, the freedom to choose patients was hampered by a sense of duty and responsibility regarding patients with psychosocial difficulties or work problems.

*“We cannot follow too many complicated (psychosocially) patients… but I think that ethically, we have to accept to do it, it is our duty as doctor in a way but we still have some flexibility regarding the intensity of such management, the number of such patients we are going to follow” FG6 line 242*

*“If the right paper is not done at the right moment, did not reach the right person, we become responsible for patients stumbling into social misery” FG5 line 561*

### Adjustment: Informal and unplanned

Adjustment strategies were essentially more informal than formal for all professional dimensions and focused essentially on buddy system and word of mouth. For medical tasks, both informal approaches, structured and continuous training in local hospitals or health institutions were combined. Participants’ resourcefulness, the fact they had a large variety of training activities and diverse work experiences were described as the most valuable elements to face such transition and develop their new roles.

*“In this job, there is the need to get this assurance, we have to know how to manage the hyper-diversity, this continuous novelty… what reassures is all these different experiences that I had, if I had done only this or that, I think I would not have the same comfort, well, diverse populations, diverse fields, different places, different people I met, all this has helped me most” » FG6 line 397*

## Discussion

Our findings indicate that recently established French-speaking primary care physicians in Switzerland faced transition challenges related to dimensions such as performance/tasks/competencies, relationships, role clarification. Participants seemed to be sufficiently prepared to manage most medical problems especially if previously exposed to both outpatient academic and private practice experiences, yet insufficiently trained in some specialties and generic tasks.

Performance is recognized as an important element in any job transition process [14, 18]. Postgraduate primary care training is less practice-oriented in Switzerland than in most European countries. Indeed, vocational training in private medical practices is limited in Switzerland because of financial issues and availability of training positions [32]. Most ambulatory training in primary care still takes place in the primary care clinics of academic medical centres. Despite these differences, our results regarding medical competencies were not very different from those reported in other European countries: newly UK qualified primary care physicians also reported insufficient training in dermatology, psychiatry, ENT [24]. This may be a source of concern since musculosketal, ENT, skin and psychosocial problems are among the most frequent health problems managed by primary care physicians [33]. Yet, the fact that the referral rate to specialists remains low for these categories of problems and lower than in other countries suggests that Swiss primary care physicians can still feel competent enough to manage most of these [34]. Our results are also in line with studies from other countries indicating that newly qualified physicians feel unprepared for non-clinical tasks requiring business/office management and leadership skills and for medico-legal issues such as long-term unemployment [2, 3, 20, 22, 28]. Such findings are not limited to primary care since new hospital consultants face similar challenges, especially in relation to financial and administrative aspects [21]. It shows that although previous work experience in a similar setting may facilitate the development of a task competence [18], it is not sufficient to develop a sense of confidence unless explicit attention is given to it during training. This is of importance since deficiencies in non-clinical competencies are correlated with emotional distress [12]. The need for a more systematic focus on management and organizational issues in medical education was recently underlined [35].

Establishing successful and satisfying work relationships within a new working environment is recognized as critical in any transition because it facilitates task performance [8, 14, 18]. Most physicians reported not having anticipated the importance of creating working relationships with “a new professional family” when setting up a new practice. They felt lost, unless they took over a medical practice and were mentored by the retiring physician or joined a large outpatient clinic also providing specialised care. Because most of our participants did not work in solo practices, feeling of isolation related more to lack of network with medical specialists than with other peers. We did not find similar reports in the literature but this can partly be explained by the fact that in several countries specialists practice in hospital settings and not in private or independent settings. Little was said about collaboration with other health professionals such as nurses, physiotherapists, pharmacists, dieticians or social workers outside the practice. The importance given to good inter-professional collaboration is rather new in Switzerland where patient-centered medical homes are only emerging. Thus it is possible that newly qualified primary care physicians did not consider such collaboration as important in the first years of their new professional role.

Clarifying roles and setting realistic expectations in a new working environment are other critical tasks of socialization and organizational changes [18]. According to situated learning theories, learning is not only about the accumulation of knowledge and skills but also the development of a new identity as member of a particular community of practice [9, 36].The experienced tensions between participants’ professional and personal values and the reality of practice seem to arise as they endorse new roles and functions, facing the need to clarify, accept and internalize new values and norms. Finding a balance between several conflicting values or issues such as altruism and self-interest involves negotiation and compromises; it can be source of stress and anxiety but also participate to the developmental process of their new professional identity [37]. In this study, sources of tension evolved around work-life balance, social responsibilities, knowing limits, practice business management. Such issues, which represent typical everyday life ethical issues, usually receive little attention among students or even junior doctors [38, 39]. Awareness about such issues should definitely be raised as junior doctors move further into the community of practitioners and before they enter independent practice.

Similarly to previous studies, much of the learning in dimensions such as tasks/performance, relationship and roles took place in the workplace, mainly informally and without much anticipation, especially for management issues [28, 40].It is known that the majority of the learning in the workplace is informal and involves learning from other people and from personal experience [41]. However, these adjustment strategies, essentially intuitive and post-hoc, may make the experience of transition even more difficult [42, 43]. Future primary care physicians would benefit from formal training and closer supervision/mentoring in some of these dimensions.

In order to support the reality of transition, greater emphasis should be set on learning in the environment for which physicians are being prepared [3], especially when physicians know more precisely in which type of context and office they intend to work. Unlike most European countries but similarly to the United States, Swiss primary care training can occur in academic hospital-related primary care settings and/or private practices. Training in academic centers allows trainees to follow chronically ill patients whose medical problems are often complicated by cultural, social and economic challenges [44–46]. Junior doctors learn to manage complex patients under close supervision. However, they may not be exposed to the true varied case mix of primary care and to the need to make decisions independently. On the other hand, training exclusively in private practice settings may also be insufficient since junior doctors tend to see more patients with minor disorders and less complex patients [47].

More training should take place in community primary care settings to facilitate such transition. We think that combining part time in the academic center over 1–2 years (in order to experience longitudinal care, medico-social and cultural complexity, formalised inter-professional collaboration) and part time for shorter periods of time in different types of community-based practices may better prepare physicians for their future practice. Experiencing different practices is useful to gain more insight into business skills for independent practice and discover different ways of endorsing management and leadership roles [7, 31]. It also better prepares residents to the case mix of general practice [22]. However, increasing the experiences of outpatient care should go together with a closer mentoring and supervision [3, 28], especially regarding issues for which newly qualified doctors feel less prepared and which may vary according to the type of setting they plan to work in. Finally, for physicians planning to open a practice in a rural area, additional formal training in pediatric and gynecological settings is warranted.

The study has several weaknesses. First, obtaining physicians’ participation into the study was challenging and we had a high rate of refusal or absence of answer. As a result, except for Geneva where the physicians’ sample was representative of the different types of practices, the sample was a convenience sample. Yet our sample covered a large variety of practice types and we reached a point of saturation after 5 focus groups. Our study is limited to the French-speaking part of Switzerland but we do not expect major differences of perceptions among physicians working in the German or Italian part of the country since training conditions are similar. Finally, although the transcripts revealed some gender issues, the sample size and the data collected did not allow for further exploration of the role of gender during such transition.

## Conclusion

Swiss primary care physicians who recently set up private practice felt insufficiently prepared in some clinical fields, for non-clinical tasks as well as for networking with specialists. They face strong tensions between personal and professional values, and the reality of practice. Transition to private practice depends not only on academic and career preparedness, but also on personal and transferable skills, such as communication, emotional management, planning, and self-confidence. Revising the postgraduate training to integrate all these skills is a challenge that Swiss primary care urgently needs to face. Similarly to other countries, Swiss postgraduate training in primary care should be further improved to better support the reality of this transition by: - placing more emphasis on leadership and management tasks and some clinical tasks in subspecialties (ENR, dermatology, rheumatology); − increasing opportunities to learn and teach in the environment for which physicians are being prepared, especially during the last year of training; − supporting their ability to take care of themselves, managing stress and ethical conflicts in order to increase satisfaction at work.

## Abbreviations

DMH: Dagmar M. Haller
ENR: ear nose throat
EU: European Union
FMH: Swiss Medical Association
JS: Johanna Sommer
MCA: Marie-Claude Audétat
MS: Melinee Schindler
NJP: Noelle Junod Perron
SM: Sanae Mazouri
UK: United Kingdom
